# Necrology

**Published:** 1902-11

**Authors:** 


### NECROLOGY.

J. R. Petty, D. V. S., died in Greensboro, N. C., August 15. Dr. Petty was operating on a horse for stringhalt when he was thrown, sustaining injuries from which he died. He was a graduate of the National and Western Veterinary Colleges, and was active in association matters in his own State, and filled acceptably the rôle of Secretary of the North Carolina Veterinary Medical Association.

